# Kinematic Changes throughout Childhood in Youth with Cerebral Palsy: Influence of Age and Orthopaedic Surgery

**DOI:** 10.3390/children11101240

**Published:** 2024-10-15

**Authors:** Nancy Lennon, Chris Church, Daniel Wagner, Tim Niiler, John Henley, Freeman Miller, Michael Wade Shrader, Jason J. Howard

**Affiliations:** Department of Orthopaedics, Nemours Children’s Health, Wilmington, DE 19803, USA; nancy.lennon@nemours.org (N.L.); dwagne06@villanova.edu (D.W.); tim.niiler@gmail.com (T.N.); john.henley@nemours.org (J.H.); freeman.miller@nemours.org (F.M.); wade.shrader@nemours.org (M.W.S.); jason.howard@nemours.org (J.J.H.)

**Keywords:** gait, kinematics, cerebral palsy, multilevel surgery

## Abstract

Background: Abnormal gait kinematics are common in youth with cerebral palsy (CP), but prior studies have not analyzed their longitudinal change throughout childhood. This study examines how age and orthopaedic surgery influence gait kinematics throughout childhood in those with ambulatory CP. Methods: In this institutional review board-approved prospective cohort study, children with spastic CP (GMFCS I–III) were recruited at age 17–40 months. Instrumented gait analysis was performed at 3-year intervals from age 4 to 21 years, collecting longitudinal kinematic data in bare feet at a self-selected speed. The change in Gait Profile Score (ΔGPS) between each pair of gait analyses (intervals) was analyzed by age distribution (<10, 10–15, ≥15 years) and by presence/absence of orthopaedic surgery. Results: The study included 31 children (GMFCS: I [13], II [14], III [4]). A baseline instrumented gait analysis was performed at age 5.8 ± 1.6 years with subsequent analysis at 2.5 ± 1.3-year intervals. Examining ΔGPS from baseline to final outcome, 87% of limbs were improved/unchanged; 298 intervals of ΔGPS were analyzed and classified as nonsurgical or surgical. Analysis revealed greater GPS improvement in intervals with surgery versus intervals without (*p* = 0.0004). Surgical intervals had significantly greater GPS improvement in the <10- vs. >15-year-old groups, *p* = 0.0063. Conclusions: Improvement in gait kinematics in children with CP is significantly influenced by age and timing of orthopaedic surgical intervention for gait correction, and was most pronounced for children <10 years old. Although surgery was associated with improved outcomes in all age groups, these improvements were significantly less for children >10 years old. These results reinforce the importance of considering the timing of orthopaedic surgery.

## 1. Introduction

Cerebral palsy (CP) is an umbrella term describing the sequelae secondary to a perinatal insult to the developing brain resulting in a permanent disorder of movement and posture [1]. Cerebral palsy is the most common pediatric neurologic disorder, and ambulatory children with a spastic motor type may exhibit gait deviations involving the sagittal plane (e.g., crouch, jump) with or without rotational malalignment [2,3,4]. These gait patterns can be associated with pain, loss of mobility, and decreased quality of life [1,2]. Preventing and minimizing this adverse natural history is one of the primary goals of orthopaedic care for children with CP [5].

Multilevel surgery (MLS) guided by instrumented gait analysis (IGA) is often performed for ambulatory children with CP to address musculoskeletal deformities affecting gait during a single surgical and rehabilitation episode [4]. The MLS approach has become the standard care for children with CP, with most prior research demonstrating short-term effectiveness [2,6,7,8] and a growing body of literature demonstrating positive long-term effects on gait [9,10,11,12]. However, research evaluating longitudinal changes in gait patterns without intervention are sparse [3]. One study reporting the results of 28 ambulatory children with CP described that gait deviations deteriorated without orthopaedic surgical intervention over a 4.4-year follow-up period (age 7.8 to 12.2 years) [3].

Understanding gait progression over childhood is essential to determining the optimal timing of surgical intervention and evaluating outcomes of treatment in youth with CP. The Gross Motor Function Measure (GMFM) and Gross Motor Function Classification System (GMFCS) have helped to define natural history of gross motor function [13,14,15,16,17,18], while other studies reported the natural history of changes in passive range of motion and foot deformities in CP [3,19,20,21,22]. Current research suggests that functional mobility and gait mechanics improve in early childhood but often regress during adolescence [2,19,23]. With this expected decline, any positive impact secondary to orthopaedic surgery may be cloaked by the declines that occur within the same time span [24]. The purpose of this study was to analyze kinematic changes over the course of childhood in ambulatory youth with CP to determine the influence of age and orthopaedic surgery on kinematic outcomes.

## 2. Materials and Methods

This was a single-center institutional review board-approved prospective cohort study. Children with unilateral or bilateral spastic CP (GMFCS I-III) were recruited in a convenience sample. Instrumented gait analyses (IGA) were performed at approximately 3-year intervals, between ages 4 to 21 years, and longitudinal kinematic data were collected and analyzed. Additional gait analyses were performed according to clinical discretion (e.g., functional changes and/or indicated for planning MLS/single-level orthopaedic surgery). For this analysis, children were excluded if they did not have an initial IGA before their 11th birthday and if they did not have a final IGA after their 13th birthday. Participants received standard clinical care throughout the study, including orthopaedic surgery and/or nonoperative treatments. Participants’ demographics and details of operative/nonoperative interventions were extracted from hospital electronic medical records.

### 2.1. Surgical Procedures

The indication and selection of orthopaedic surgical procedures for patients were determined as part of standardized clinical care at our center using objective measurements of data from IGA, physical examination, and radiological findings. Prior studies document the technical details of the operative procedures [7,25,26,27].

### 2.2. Instrumented Gait Analysis

Kinematic data were collected between 2002 and 2022 using IGA and an eight- or 12-camera motion capture system (Motion Analysis, Santa Rosa CA or Qualisys, Göteborg, Sweden) with participants walking at a self-selected speed (barefoot) with a preferred walking aide as needed. Thirty-four retroreflective markers were placed on anatomical landmarks according to the Cleveland Clinic marker set [28].

The primary outcome variable was the change (Δ) in the Gait Profile Score (GPS) [29]. The GPS has been proven to be a validated measure of gait pattern abnormality [29,30]. It provides a single index measure that summarizes deviations of kinematic gait data relative to normative data. Improvement in gait pattern is represented as a negative ΔGPS. We defined the ΔGPS from the first and last visits as the participants’ global kinematic outcome. Change in GPS was also analyzed between consecutive IGA visits to examine the effects of age and surgery on change.

The GPS were also placed into nine Gait Variable Scores (GVS) representing kinematic components derived from the hip (rotation, abduction, and flexion), pelvis (obliquity, tilt, and rotation), knee (flexion), and foot/ankle (dorsiflexion and foot progression angle) [29,30]. The GVS were examined to identify segmental contributions to overall changes in gait kinematics.

### 2.3. Statistical Analysis

Utilizing the minimal clinically important difference (MCID) for the GPS (±1.6°) [31], we examined change over childhood in GPS from baseline to final visits, categorizing children as improved, unchanged, or worse within each GMFCS level. All affected children’s limbs in the study sample we evaluated and reported separately for all statistical analyses.

Change in GPS was determined for each interval between two consecutive IGA events and stratifying children into three groups according to age at the second IGA comprising intervals of <10, 10–15, and >15 years. Intervals between IGA visits were also stratified by presence/absence of intervening surgical intervention (surgical/nonsurgical). Change in GPS was compared by age and surgical intervention groups using analysis of variance with Tukey honestly significant difference post hoc analyses.

Changes in component GVS (ΔGVS) were compared with zero (no change) using Welch’s *t*-tests after surgical intervals, stratified by age group. For those limbs exhibiting a deterioration in GPS, ΔGVS components from first to last IGA visits were compared with zero (no change) using one-sample *t*-tests. Significance levels were determined by the Bonferroni correction. Statistical analysis was completed using R version 4.0.3 [32].

## 3. Results

Thirty-one participants (62 lower limbs: 47 [76%] affected, 15 [24%] unaffected) met the inclusion criteria. Twenty-three (74%) individuals were male and eight (26%) were female. Sixteen (52%) of the individuals had bilateral CP while 15 (48%) had unilateral CP, represented by GMFCS levels I (13, 42%), II (14, 45%), and III (4, 13%). There were 180 IGA visits for the entire cohort creating 298 single limb intervals available for analysis. Of these intervals, 235 were nonsurgical and 63 were surgical. The mean age at first and last IGA was 5.8 ± 1.6 and 17.8 ± 2.1 years, respectively. The mean number of IGA performed for each participant was 5.8 ± 1.6, with a mean time between visits of 2.5 ± 1.3 years. Time from surgery to postoperative evaluation was 1.7 ± 1.0 years (range 0.5 to 4.6 years).

For the entire cohort, 221 orthopaedic procedures (59 bony/162 soft tissue) were performed within 41 discrete multilevel surgical events for 25 patients (81%), the majority of which were for lower limb correction (Table 1).

**Figure 1:**
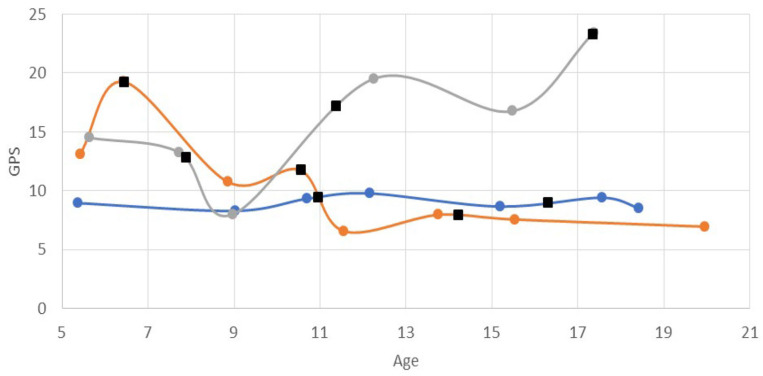
Graphical representation of kinematic trajectories from first to last instrumented gait analysis visit by ΔGPS (improved [orange], unchanged [blue], deteriorated [grey]). Surgical events are denoted ().GPS variability within trajectories were common, with surgical events having a significant influence (see text). ΔGPS: change in Gait Profile Score.

**Figure 2:**
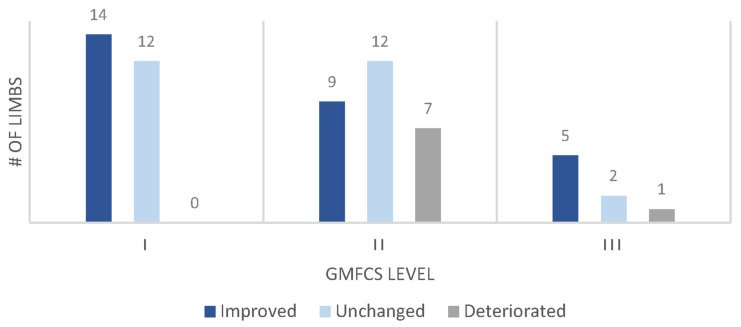
Breakdown of individual lower limb kinematic change (ΔGPS from first to last instrumented gait analysis visit) by GMFCS level. These outcomes were determined using the minimal clinically important difference for Gait Profile Score (improved: ΔGPS < −1.6°; unchanged: −1.6° ≤ ΔGPS ≤ 1.6°; deteriorated: ΔGPS > 1.6°). GMFCS: Gross Motor Function Classification System, ΔGPS: change in Gait Profile Score.

Figure 1 presents an example of kinematic trajectories for three different patients (limbs), showing gait pattern changes throughout childhood, including after surgical events. Comparing the ΔGPS to MCID between the baseline and final IGA visits, 87% of the limbs were improved (*n* = 28, 45%) or unchanged (*n* = 26, 42%) while the remaining eight (13%) deteriorated (Figure 2). With respect to surgical treatment throughout childhood, ΔGPS was significantly higher in surgical intervals compared with nonsurgical intervals (−1.4° ± 2.9°, *n* = 63 limbs vs. 0.1° ± 2.4°, *n* = 235; *p* = 0.0004). Stratified by GMFCS, none of the limbs in GMFCS I experienced ΔGPS deterioration compared with GMFCS levels II (*n* = 7, 25.0%) and III (*n* = 1, 12.5%) (Figure 2).

**Figure 3:**
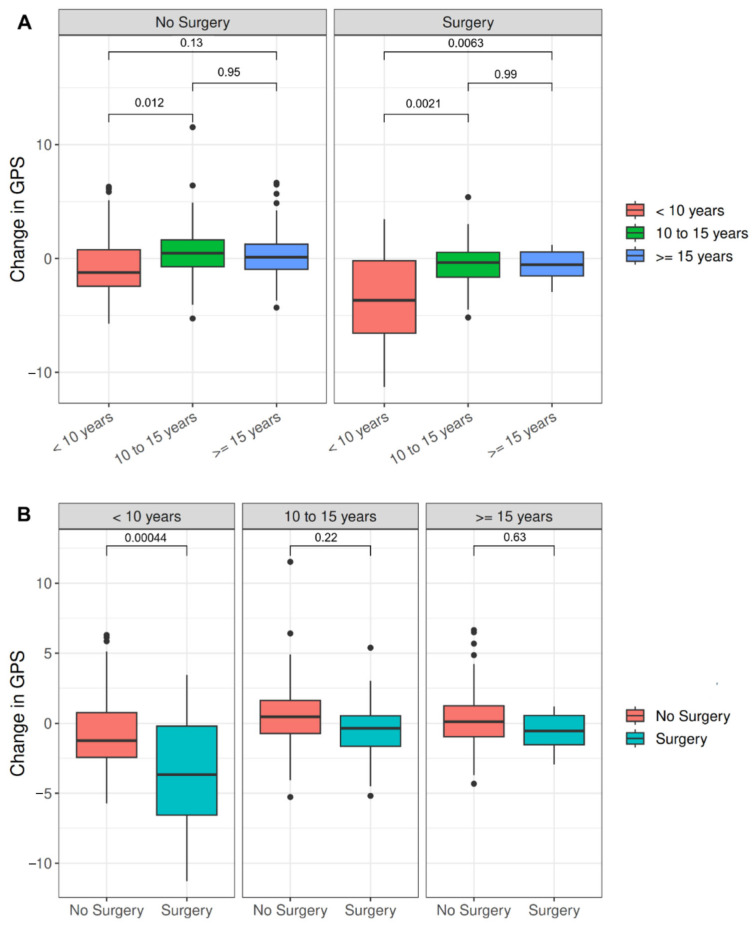
(**A**) ΔGPS box plots stratified by surgical interval and age. (**B**) ΔGPS box plots stratified by age group and surgical interval. For each box, the horizontal line indicates the median and its top and bottom the interquartile range. Whiskers and individual dots represent the overall range and outliers, respectively. ΔGPS: change in Gait Profile Score. No Surgery, <10 *n* = 72, 10–15 *n* = 83, >15 *n* = 80, Surgery <10 *n* = 16, 10–15 *n* = 27, >15 *n* = 20.

For the entire cohort (Figure 3A), no significant ΔGPS was seen for any age group in nonsurgical intervals, but there was a significant difference in ΔGPS for the <10 vs. 10–15 years of age groups (−0.7° ± 2.7° vs. 0.6° ± 2.4°, *p* = 0.012). By contrast, for the surgical intervals, a significant GPS improvement was seen for the <10 vs. 10–15 years of age groups (*p* = 0.0021) and for the <10 vs. >15 years of age groups (−3.6° ± 4.0° vs. −0.7° ± 1.3°, *p* = 0.0063). No significant differences in ΔGPS were seen between the two older age groups. Within age stratifications (Figure 3B), ΔGPS was significantly higher in surgical vs. nonsurgical intervals in the <10 years of age group (−3.6° ± 4.0° vs. −0.7° ± 2.7°; *p* = 0.00044), but no differences were found for the older groups.

**Figure 4:**
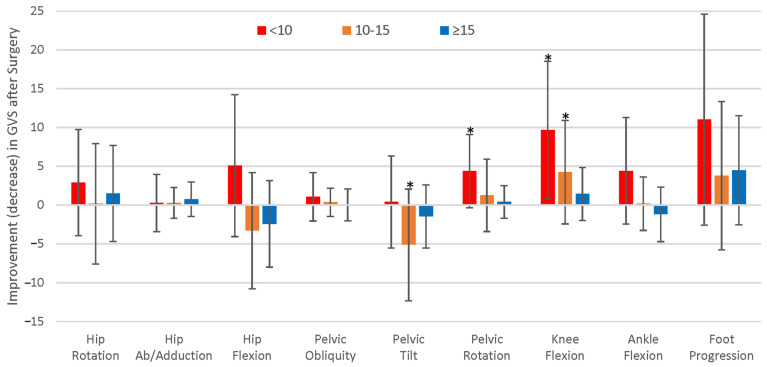
ΔGVS after surgical intervals by age group. Bars represent the mean and error bars are the standard deviation. * *p* < 0.05. Note the significant improvements in GVS for the <10 years of age group. ΔGPS: change in Gait Profile Score, GVS: Gait Variable Score.

Closer examination of surgical intervals was performed by analyzing change in GVS components (Figure 4). Significant GVS improvements were seen for pelvic rotation (adjusted *p* = 0.033) and knee flexion (adjusted *p* = 0.0081) in the <10 years of age group and for knee flexion (adjusted *p* = 0.036) in the 10–15 years of age group. Significant GVS deterioration was seen for pelvic tilt in the 10–15 years of age group (adjusted *p* = 0.014). For the eight limbs that deteriorated over the course of childhood, the primary contributors to the ΔGPS were increases in anterior pelvic tilt (adjusted *p* = 0.034) and hip flexion (adjusted *p* = 0.00021).

## 4. Discussion

Studies investigating longitudinal changes in gait kinematics over childhood are sparse, with most being retrospective, having short-term follow-up, and mainly limited to the assessment of treatments. Understanding the trajectory of kinematic change, with and without orthopaedic surgery, is important to understand trends in the natural history of gait development and influence of surgical interventions on outcomes. In pursuit of these goals, our prospective study of children with ambulatory CP documents the longitudinal change in global gait patterns, the primary contributing kinematic variables responsible for overall gait pattern, and the responsiveness to surgical interventions from early childhood to the late teens.

The importance of regular orthopaedic follow-up combined with the use of IGA to prevent loss of gait function has been emphasized [19]. In the study by Haumont and colleagues, timely application of orthopaedic surgery guided by IGA was associated with positive gait outcomes, including significant improvements in Gait Deviation Index (GDI), GMFM, gait speed, knee extension, and foot progression angle [19]. The GPS, similar to the GDI, provides a quantitative assessment of overall gait kinematic severity that is correlated with motor function and with the GDI [33,34]. Others found similar improvements after MLS, with a −4.6° ΔGPS 12 months postoperatively reported by Thomason et al. [7] and −4.3° by Rutz et al. [35]. In our study, GPS for the entire cohort was improved or maintained in 87% of limbs, most notably after surgical interventions and for the youngest children (<10 years old). Although postoperative improvement in gait for the current study was evident, our cohort overall had less GPS improvement. This could be explained by the wider age range in our study, given that our youngest age group had similar levels of improvement to these reports of relatively young cohorts. Taken together, these results highlight the benefits of orthopaedic surgery for ambulatory CP, with the caveat that the goal for older children may be best represented through maintenance of gait rather than the absolute improvements seen in children under age 10 years. With respect to timing of surgery, however, increased risks of recurrence of muscle contractures reported for children under age 7 years should be considered when determining the optimal age range for MLS [36].

A substantial number of participants in the current study had intervals between IGA assessments without surgery, allowing for at least a partial assessment of natural gait history for youth with ambulatory CP. Bell and colleagues [3] reported deterioration of temporospatial parameters and kinematics in a nonsurgical group with short follow-up, finding significant reductions in hip extension during stance, peak knee flexion during swing, and ankle dorsiflexion over time (4.4 years from age 7.8 to 12.2 years). In agreement with these findings, we found no significant improvements in GPS for children older than 10 years in the current study. By contrast, we did find significant improvements in gait between IGA visits for children under age 10 years. This may be explained by increases in gross motor function expected for children classified as GMFCS levels I to II (and to some extent III) during early- and mid-childhood [16]. The lack of improvement in GPS seen in the older age groups is consistent with plateauing of GMFM curves in the preteen and teenage years, coupled with reported deterioration in lower extremity joint passive range of motion with growth [16,22]. These same factors could explain the minimal effect on gait outcomes postoperatively for the older age groups in the current study.

Age had a significant impact on kinematic changes. During early childhood, concurrent with rapid gross motor functional development [16,17,19], we found that gait kinematics were more likely to improve compared with older age intervals during both nonsurgical and surgical intervals. There are several potential reasons why surgery had less effect in older children. The GPS is highly sensitive to changes in the sagittal plane, and orthopaedic surgery is more commonly directed at gait correction in this plane for younger children. Torsional correction of long bones, however, is more commonly performed for older children, and the axial plane changes have less representation in the GPS. Additionally, surgical goals often differ in the teen years, focusing on musculoskeletal problems such as improving foot position and/or reducing pain rather than correction of overall gait kinematics.

Analysis of GVS has been provided in several studies [35,37,38,39,40]. Rutz et al. looked at changes in GVS after single-event multilevel surgery, demonstrating the effects of preoperative GPS, age, and previous surgery on outcomes [35]. In agreement with these results, improvements in GVS for the current study were most significant for the youngest age group, most notably at the pelvis and knee. Although children over 10 years of age demonstrated some improvements at the knee, this was overshadowed by substantial deterioration in hip flexion and anterior pelvic tilt (Figure 4). This may follow the treatment philosophy adopted at our institution for performing early soft-tissue muscle lengthening surgeries, followed by long bone osteotomies and foot correction surgery in the early teen years to improve alignment and prevent worsening of compensatory mechanisms that often progress with age. Although our study showed little improvement in GPS for the older age groups, few patients deteriorated, which was viewed as a relatively positive outcome compared with the risk of gait decline during adolescence reported in the literature [8]. This lack of gait deterioration may be a result of close monitoring via regular orthopaedic follow-up, with timely surgical intervention when gait deterioration was identified.

The strengths of the current study lie in its prospective study design and the long follow-up with multiple IGA events through the course of childhood. Despite these aspects, there are several limitations. This was a small convenience sample of individuals seen at our facility for orthopaedic care over a multi-year period, and thus it is not population-based. The lack of a control group limits the ability to discern the natural history of gait over time; however, there were several patients who did not undergo any surgical procedures for gait correction. In addition, there was substantial heterogeneity in gait deviations and surgical prescriptions–types and timing–for correction. We also did not precisely control the timing of IGA visits in relation to the dates of surgical intervention.

## 5. Conclusions

Global change in gait kinematics in children with CP are greatly influenced by age and surgical intervention. Improvements in gait are more likely in early vs. late childhood. Surgeries in the 10–15 and >15 age intervals showed less change in patients’ GPS scores, yet gait pattern was largely maintained over the longer term. Orthopaedic surgery had a positive impact in all three age groups, with the greatest postsurgical changes seen in children under age 10 years. The results of this longitudinal prospective study may be used to help inform the best timing for gait correction surgery in bilateral CP. Information gleaned regarding the positive role of orthopaedic surgery on gait may be imparted during parent/patient preoperative counseling to help in decision-making. 

## Figures and Tables

**Table 1:** Type and frequency of bone/soft-tissue procedures.

Bony Procedures	*n*	Soft-Tissue Procedures	*n*
Femoral osteotomy	12	Hamstring lengthening	47
Calcaneal lengthening	12	Gastrocnemius lengthening	40
Tibial osteotomy	9	Achilles tendon lengthening	18
Metatarsal osteotomy	8	Adductor lengthening	16
Akin osteotomy	6	Rectus femoris transfer	11
Medial column correction	4	Tibialis anterior transfer	7
Cuneiform osteotomy	3	Iliopsoas lengthening	6
Distal femoral extension osteotomy	2	Knee capsulotomy	5
Pemberton osteotomy	1	Tibialis posterior lengthening	4
Dega pelvic osteotomy	1	Tibialis posterior transfer	3
Cuboid osteotomy	1	Plantaris tenotomy	3
		Patellar plication	2
Total	59	Total	162

## Data Availability

The data presented in this study are available on request from the corresponding author due to privacy restrictions.
